# Curcumin Analogs Reduce Stress and Inflammation Indices in Experimental Models of Diabetes

**DOI:** 10.3389/fendo.2019.00887

**Published:** 2019-12-18

**Authors:** Saumik Biswas, Shali Chen, Guang Liang, Biao Feng, Lu Cai, Zia A. Khan, Subrata Chakrabarti

**Affiliations:** ^1^Department of Pathology and Laboratory Medicine, Western University, London, ON, Canada; ^2^Chemical Biology Research Centre, School of Pharmaceutical Science, Wenzhou Medical University, Wenzhou, China; ^3^Department of Pediatrics, Pediatric Research Institute, University of Louisville, Louisville, KY, United States

**Keywords:** diabetic complications, curcumin analogs, inflammation, angiogenesis, fibrosis

## Abstract

Chronic inflammation and oxidative stress lead to a multitude of adverse cellular responses in target organs of chronic diabetic complications. Curcumin, a highly investigated phytochemical, has been shown to exhibit both anti-inflammatory and antioxidant activities. However, the clinical application of curcumin has been greatly limited due to a poor pharmacokinetic profile. To overcome these limitations, we have generated analogs of curcumin to enhance bioavailability and offer a preferable pharmacokinetic profile. Here, we explored the effects of two mono-carbonyl curcumin analogs, L2H21 and L50H46, in alleviating indices of inflammation and oxidative stress in cell culture and mouse model of diabetic complications. Our results show that L2H21 and L50H46 normalize inflammatory mediators (*IL-6* and *TNF-*α), extracellular matrix proteins (*FN* and *COL4*α*1*), vasoactive factors (*VEGF* and *ET-1*) and a key transcriptional coactivator (*p300*) in cultured human retinal microvascular endothelial cells (HRECs) and dermal-derived microvascular endothelial cells (HMVECs) challenged with high levels of glucose. These curcumin analogs also reduced glucose-induced oxidative DNA damage as evidenced by 8-OHdG labeling. We further show that treatment of streptozotocin-induced diabetic mice with curcumin analogs prevents cardiac and renal dysfunction. The preservation of target tissue function was associated with normalization of pro-inflammatory cytokines and matrix proteins. Collectively, our results show that L2H21 and L50H46 offer the anti-inflammatory and antioxidant activities as has been reported for curcumin, and may provide a clinically applicable therapeutic option for the treatment of diabetic complications.

## Introduction

As the incidence of diabetes rises globally, the risk of sustaining considerable organ damage and developing secondary complications remain at an all-time high. Vascular endothelial cells represent the primary target of metabolic insults in diabetes (1). High levels of glucose in diabetes induce multiple biochemical pathways in vascular cells, including increased oxidative stress, generation of advanced glycation end-products, excess synthesis of extracellular matrix, altered protein kinase C activity, increased vascular permeability, and endothelial cell apoptosis (2, 3). Of these, increased oxidative stress perhaps plays the key pathogenetic stimulus for the genesis of diabetic complications (4). Increased oxidative stress degrades and sequesters nitric oxide, causing an imbalance in vasoconstrictors and vasodilators and leading to impaired vasoregulation—the hallmark of endothelial dysfunction (5, 6). Oxidative stress also activates the nuclear factor-κB (NF-κB) signaling pathway, inducing the production of proinflammatory cytokines (7, 8). Inflammatory cytokine production through NF-κB requires the transcription co-activator p300 (9, 10). We and others have additionally shown that glucose-induced upregulation of p300 can regulate the expression of genes for extracellular matrix (ECM) proteins and vasoactive factors in endothelial cells (11, 12). Therefore, these studies underscore the discovery and development of therapeutic agents that combat diabetes-induced oxidative stress and inflammatory responses.

One highly investigated dietary phytochemical is curcumin, the principal curcuminoid of turmeric (found in the *Curcuma longa* plant), which is used as a dietary pigment and spice in the Indian subcontinent and China (13). In these regions, curcumin has also been used for many centuries as a therapeutic agent in treating inflammatory ailments (14). Adding to the possible roles of curcumin as an anti-inflammatory, anti-oxidative, anti-fungal and anti-bacterial agent (14–16), our laboratory has previously demonstrated that curcumin is capable of preventing the effects of glucose-induced functional and structural changes at both the cellular and organ levels (17–19). Specifically, the application of curcumin is effective in reducing the degree of glucose-induced oxidative stress in endothelial cells and in the heart of diabetic animals (17, 18). Similarly, short-term treatment of curcumin significantly attenuates diabetes-induced levels of vasoactive factors (endothelin-1 and eNOS), TGF-β1, ECM proteins (fibronectin), and oxidative stress markers (heme oxygenase-1, nitrotyrosine, and 8-OHdG) in kidneys (19). We have further shown that the effects of curcumin are largely mediated through the inhibition of p300 and NF-κB (19).

Despite the plethora of *in vitro* and *in vivo* mechanistic studies on the therapeutic actions of curcumin, translating these experimental findings into clinical applications have been increasingly difficult due to the poor bioavailability and rapid metabolism of curcumin (20, 21). To overcome this limitation, our group has synthesized novel analogs of curcumin (22, 23). Two of these analogs, L50H46 and L2H21, have shown biological activity comparable to curcumin but enhanced pharmacokinetic profiles.

The aim of the present study is to evaluate the efficacy of L50H46 and L2H21 on vascular endothelial cells and on the development of functional tissue deficits characteristic of diabetic complications. Our results show that L50H46 and L2H21 prevent cardiac and renal dysfunction in a mouse model of diabetes. These activities were comparable to curcumin treatment of diabetic mice. We also found that preservation of tissue function is associated with normalized expressions of inflammatory mediators, extracellular matrix proteins, and *p300*. We confirmed these results in cultured vascular endothelial cells challenged with high levels of glucose, showing normalization of inflammatory and oxidative stress marks in endothelial cells by curcumin analogs. Collectively, our results show that both L50H46 and L2H21 curcumin analogs may provide therapeutic benefit in preventing chronic diabetic complications.

## Materials and Methods

### Cell Culture

Human retinal endothelial cells (HRECs) and dermal microvascular endothelial cells (HMVECs) were obtained from Cell Systems (Walkersville, MD, USA) and Lonza (Kirkland, WA, USA), respectively. Cell culture conditions used in this study have been previously described (24–26). Briefly, cells were cultured using endothelial basal media-2 (EBM-2, Lonza), supplemented with endothelial growth media-2 (EGM-2) SingleQuots (Lonza Inc.), in 25 cm^2^ tissue culture flasks and maintained in a humidified atmosphere containing 5% CO_2_ at 37 °C. At 80% confluence, cells were cultured in serum- and growth factor-free medium overnight before exposure to different D-glucose levels (5 mmol/L, mimicking normal glucose [NG] concentrations, or 25 mmol/L, mimicking high glucose [HG] concentrations). The control media formulation contains approximately 5.5 mM glucose (100 mg/dL), which approximates to normal blood glucose levels *in vivo*. In cell culture studies, concentrations of glucose approaching 10 mM (180 mg/dL) are considered pre-diabetic and concentrations above 10 mM are analogous to diabetic conditions. To investigate the effect of curcumin analogs, cells were pre-treated with L2H21 or L50H46 for 1 h before exposure to high glucose for 48 h. Concentrations of L2H21 and L50H46 were selected following cell viability assessment. In brief, concentrations that did not reduce cell viability were used. All *in vitro* experiments were independently repeated at least three times and performed with 6 replicates, unless specified.

### Animal Model

Male C57BL/6 mice (8 weeks of age) were obtained from Charles River (Montreal, QC, Canada) and randomly divided into five weight-matched groups (*n* = 6/group): non-diabetic control (C), diabetic (D), and diabetic mice treated with either curcumin (D-Cur), L50H46 (D-L50H46), or L2H21 (D-L2H21). Mice were made diabetic by intraperitoneal injection of multiple, low-dose streptozotocin (STZ; Sigma-Aldrich, St. Louis, MO, USA; 50 mg/kg, dissolved in citrate buffer, pH = 5.6) (18, 26–28). Non-diabetic control mice received citrate buffer alone. Diabetes was confirmed by blood glucose level >20 mmol/L on two consecutive days. Following induction of diabetes, curcumin (20 mg/kg) or its analogs (10 mg/kg) were administered daily by oral gavage in 1% sodium carboxymethyl cellulose solution (CMC-Na) for 8 and 16 weeks. Untreated diabetic mice and non-diabetic control mice were given the same volume of vehicle alone (CMC-Na). Previous studies testing the effect of curcumin in animal models of diabetes have used doses ranging from 50 to 150 mg/kg (23, 29, 30). However, our pilot studies showed that 20 mg/kg curcumin is effective in suppressing inflammatory cytokine expression in the STZ-induced diabetic mouse model. Hence, this low dose was used as a comparator for the curcumin analogs. The dose for curcumin analogs was selected based on our previous studies utilizing lipopolysaccharide-induced proinflammatory cytokine production (22), which is largely mediated through NF-κB (31). Mice were monitored and recorded for blood glucose, glucosuria, ketonuria, and body weights. Prior to euthanasia, echocardiographic analyses were performed. After 2 or 4 months of diabetes, mice were euthanized by carbon dioxide inhalation, and tissues were harvested including retinal, renal cortical, and left ventricular tissues.

### Toxicity and Histopathological Analyses

To determine potential adverse effects of L2H21 and L50H46 in mice, toxicity analyses were performed in addition to the regular monitoring of mice. Age-matched mice were divided into six groups: L2H21 high-dose group (1g/kg, *n* = 7), L2H21 middle-dose group (0.5g/kg, *n* = 7), L2H21 low-dose group (0.1g/kg, *n* = 5), L50H46 high-dose group (1g/kg, *n* = 7), L50H46 middle-dose group (0.5g/kg, *n* = 7), L50H46 low-dose group (0.1g/kg, *n* = 6). The analogs were administered only once orally, as a single-dose, and mice were followed-up for 7 consecutive days. Following this time-point, organs were excised, fixed in 10% buffered formalin solution and embedded, and sectioned into 5 μm thick sections. The tissue sections were then stained with hematoxylin and eosin (H&E) for routine histology. A blinded pathologist evaluated the histopathological damage using a light microscope and the images were captured (Nikon, Japan).

### Analysis of Cardiac Function and Size

Cardiac function was assessed in lightly anesthetized mice. Briefly, mice were anesthetized by 1.5% inhaled isoflurane through continuous flow of 2% oxygen for the induction of anesthesia (VetFlo Vaporizer; Kent Scientific, Torrington, CT, USA). Cardiac function was then assessed through echocardiography and Doppler ultrasound using a 40 MHz linear array transducer (MS-550D) and the Vevo 2100 imaging system (VisualSonics, Toronto, ON, Canada). As documented in our previous studies (27, 32), left ventricular ejection fraction (EF) was used as an index of cardiac contractile function, while mitral inflow patterns (E/A ratio) and mitral annulus velocities (E′/A′ ratio) were used to assess diastolic dysfunction. Furthermore, after mice were euthanized, the tibia length and heart weight were recorded to assess the degree of cardiac hypertrophy (33).

### Assessment of Urinary Albumin and Creatinine

Prior to euthanasia (24 h before the end-point), animals were placed in metabolic cages for 24 h and urine samples were collected from all groups. Albumin and creatinine levels were measured using commercial kits (Albuwell and Creatinine companion kit; Exocell, Philadelphia, PA, USA), and albumin-to-creatinine ratios (ACR) were determined (28, 34).

### Synthesis of Curcumin Analogs

Curcumin analogs were synthesized and characterized as described by us previously (22, 23). Additional information regarding the characteristics of the analogs is provided in Figure S1. Curcumin (Sigma; Oakville, ON, CAN) was used for comparison.

### WST-1 Assay

The effect of curcumin and its analogs on cell viability was measured using the water soluble tetrazolium salt-1 viability assay (WST-1, Roche). The assay was carried out as previously described (26, 35).

### Immunofluorescence

As previously described (33), HMVECs or HRECs were plated on eight-chamber tissue culture slides and incubated for 48 h after glucose challenge (5 or 25 mmol/L), with or without curcumin or curcumin analog pre-treatment. The cells were fixed with methanol and then stained for an oxidative DNA damage marker, 8-hydroxy-2′-deoxyguanosine (8-OHdG; 1:50, Santa Cruz Biotechnology). Fluorescein isothiocyanate (FITC)-conjugated goat IgG (Vector Laboratories, Burlingame, CA) was used for detection. Slides were mounted in Vectashield fluorescence mounting medium with 4,6-diamidino-2-phenylindole (DAPI; Vector Laboratories) for nuclear staining. Microscopy was performed by an examiner unaware of the identity of the samples using a Zeiss LSM 410 inverted laser scan microscope equipped with fluorescein, rhodamine, and DAPI filters (Carl Zeiss Canada, North York, ON, Canada).

### Enzyme-Linked Immunosorbent Assay (ELISA)

Enzyme-linked immunosorbent assays (ELISAs) were performed to measure tumor necrosis factor-α (TNF-α; R&D Systems, Minneapolis, MN, USA), fibronectin (FN; R&D Systems), collagen-IV alpha 1 chain (COL4A1; Cloud-Clone Corp., Katy, TX, USA), vascular endothelial growth factor (VEGF; R&D Systems), endothelin-1 (ET-1; R&D Systems), and P300 (Cloud-Clone Corp.) in cell or tissue lysates. Briefly, protein concentrations were assessed using the BCA protein assay kit (Pierce, Rockford, IL, USA) and 50 μg of total proteins were used for each assay. Optical density at 450 nm was measured using Multiskan FC Microplate Photometer (Thermo Fisher Scientific, Boston, MA, USA). Readings were corrected using 568 nm absorbance (26–28, 32, 34).

### RNA Isolation and Quantitative Real-Time Polymerase Chain Reaction (RT-qPCR)

Total RNA from cells and tissues was extracted using TRIzol reagent (Invitrogen, Burlington, ON, CAN), as described previously (11, 12, 17–19, 24–33, 35). Briefly, RNA was extracted with chloroform, followed by centrifugation to separate the sample into aqueous and organic phases. RNA was recovered from the aqueous phase by isopropyl alcohol precipitation and suspended in diethylpyrocarbonate (DEPC)-treated water. Total RNA (1 μg) was used for cDNA synthesis with high capacity cDNA reverse transcription kit (Applied Biosystems, Foster City, CA, USA). The resulting cDNA products were stored at −20°C until further experimentation. Transcript levels were measured using LightCycler (Roche Diagnostics Canada, Laval, QC, CAN) as previously described (11, 12, 17–19, 24–33, 35). For a final reaction volume of 20 μL, the following reagents were added: 10 μL SYBR Advantage qPCR Premix (Clontech, Mountain View, CA, USA), 1 μL each of forward and reverse 10 μmol/L primers (Table S1), 7 μL water, and 1 μL cDNA template. The standard curve method was used to quantify mRNA levels. Standard curves were constructed by using serially diluted standard templates (1:1, 1:10, 1:100, and 1:1,000). The data were normalized to a housekeeping gene, *18S ribosomal RNA* or β*-actin*, to account for differences in reverse transcription efficiencies and the amount of template in the reaction mixtures. In our pilot studies, we observed both *18S ribosomal RNA* and β*-actin* to be stable in HG as well diabetic mouse tissues. In cases where *18S ribosomal RNA* was used, cDNA samples were diluted to bring the threshold cycle of house keeping gene to levels similar to β*-actin*.

### Statistical Analysis

Data are presented as mean ± standard error (SEM), unless specified. Statistical significance was determined by using one-way ANOVA, followed by Tukey's *post-hoc* analysis, for multiple comparisons. A *P*-value of 0.05 or less was considered to be significant. All calculations were performed with GraphPad Prism 7 (La Jolla, CA, USA).

## Results

### Low Concentrations of L50H46 and L2H21 Do Not Reduce Cell Viability in Cultured Endothelial Cells

Our first objective was to establish effective and non-toxic doses of curcumin analogs in cultured endothelial cells. We utilized HMVECs and HRECs as models of microvascular endothelial cells and exposed the cells to varying concentrations of curcumin analogs. Analysis of cell viability by WST-1 assay showed that curcumin and L2H21 reduce viable cell numbers at concentrations equal to or >20 μM (data not shown). Treatment of cells with L50H46 reduced viability at concentrations equal to or >1.25 μM. Based on these results, we selected 10 μM concentration for L2H21 and 0.625 μM concentration for L50H46 for subsequent *in vitro* experiments (Figure S1D). We also tested and observed no reduction in cell viability following exposure of cells to normal (5 mmol/L) or high levels of glucose (25 mmol/L) for up to 96 h (data not shown).

### Toxicity Studies and the Effects of Curcumin Analogs on Blood Glucose Levels and Body Weights in Mice

We administered L50H46 and L2H21 in mice and examined heart, kidney, lung, and liver tissues for evidence of frank toxicity. Our results show that mice administered L50H46 and L2H21, at doses ranging from 100 mg/kg to 1 g/kg, exhibit no observable histological changes in hearts, lungs, kidneys, and liver tissues, as demonstrated in Figure S2. Although we performed toxicity studies using a single dose as done in most preclinical studies, it may not be reflective of any long-term toxicity. Therefore, based on our initial, promising results and the need to perform a long-term study to examine the effects of the analogs on measures of diabetic complications, we elected to use 10 mg/kg dose for the curcumin analogs.

We tested the effect of curcumin analogs in a STZ-induced model of type 1 diabetes. Previous studies have shown that curcumin reduces glycemia in type 2 models (36), and improves insulin resistance (37). Therefore, in type 2 diabetes, curcumin may provide protective effects by reducing the stimulus (hyperglycemia and dyslipidemia) (38). Since our primary goal is to determine whether curcumin analogs prevent hyperglycemia-induced target organ dysfunction, we used the type 1 model. Following the completion of our long-term study, we did not observe any toxic phenotypic effects in the diabetic animals administered curcumin or its analogs. Our results also show that the diabetic mice had significantly increased blood glucose levels and reduced body weights compared to non-diabetic controls, which was expected (Table S2). As desired for our study, blood glucose levels in diabetic mice treated with curcumin or its synthetic analogs were not significantly different compared to untreated diabetic mice at both time-points. In terms of body weights, the values were significantly reduced in diabetic mice treated with curcumin or L50H46 at the 2-month mark when compared to non-diabetic controls; whereas, the body weights of the L2H21-treated diabetic mice were not significantly different. Furthermore, at 4-months, both L2H21 and L50H46-treated diabetic mice demonstrated significantly improved body weights when compared to untreated diabetic and curcumin-treated diabetic mice.

### Curcumin Analogs Normalize Indices of Glucose-Induced Vascular Damage *in vitro*

**Figure 1:**
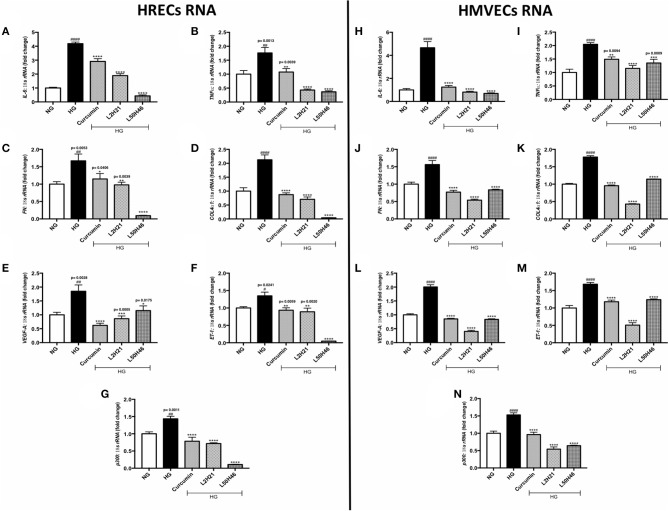
Curcumin analogs significantly reduced gene expressions associated with glucose-induced vascular damage *in vitro*. Left panel: RT-qPCR analyses showing increased mRNA levels of **(A)** *IL-6*, **(B)** *TNF-*α, **(C)** *FN*, **(D)** *COL4*α*1*, **(E)** *VEGF-A*, **(F)** *ET-1*, and **(G)** *p300* in HRECs exposed to HG for 48 h. The administration of curcumin or its analogs (1-h pre-treatment) was able to dampen the glucose-induced levels of these transcripts. Right panel: RT-qPCR analyses showing elevated mRNA levels of **(H)** *IL-6*, **(I)** *TNF-*α, **(J)** *FN*, **(K)** *COL4*α*1*, **(L)** *VEGF-A*, **(M)** *ET-1*, and **(N)** *p300* in HMVECs exposed to HG for 48 h. Similar to HRECs, HMVECs treated with curcumin or its derivatives also had significantly lower levels of high glucose-associated molecules [data expressed as a ratio to *18S ribosomal RNA* (mean ± SEM); normalized to NG; ^#^significance between NG vs. HG; *significance between HG vs. Treatment group; *^, #^*P* < 0.05, **^,*##*^*P* < 0.01, ***^, *###*^*P* < 0.001, and ****^, *####*^*P* < 0.0001; *n* = 6 from three independent experiments and performed in triplicates; NG (normal glucose) = 5 mM D-glucose; HG (high glucose) = 25 mM D-glucose; data analyzed by ANOVA followed by Tukey's *post-hoc*].

Exposure to high levels of glucose induces pro-inflammatory (26–28, 39), pro-angiogenic (11, 17, 25, 35, 40), and pro-fibrogenic (12, 24, 27, 32, 33) phenotypes in vascular endothelial cells. We selected the most prominent markers of these processes and determined the expression levels in HRECs and HMVECs under basal and high glucose conditions. In keeping with our previous studies (11, 12, 17, 24–28, 32, 33, 35, 39, 40), both cell types demonstrated significant elevations of *TNF-*α, *IL-6, FN, COL4*α*1, VEGF-A, ET-1*, and *p300* transcripts following 48-h high glucose (HG) culture (Figures 1A–G). Conversely, the administration of L2H21 and L50H46 significantly reduced these transcripts in cells exposed to HG. Specifically, in HG-treated HRECs and HMVECs, both curcumin analogs reduced *IL-6* (Figures 1A,H), *TNF-*α (Figures 1B,I), *FN* (Figures 1C,J), *COL4*α*1* (Figures 1D,K), *VEGF-A* (Figures 1E,L), *ET-1* (Figures 1F,M), and *p300* (Figures 1G,N) levels compared to HG. Curcumin, used as a comparator, showed the same results.

### The Impact of Curcumin Analogs on Diabetes-Associated Protein Markers in Endothelial Cells Cultured With High Glucose

**Figure 2:**
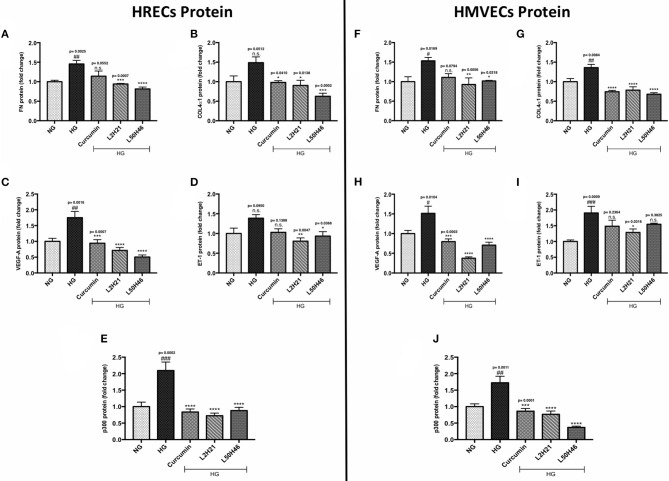
Curcumin and its analogs alter proteins associated with glucose-induced vascular damage *in vitro*. Left panel: Protein levels of **(A)** FN, **(B)** COL4α1, **(C)** VEGF-A, **(D)** ET-1, and **(E)** p300 in HRECs pre-treated with L2H21 or L50H46 before exposure to HG. Protein levels were measured by ELISA. HG increased the selected proteins in HRECs, while the curcumin derivatives significantly reduced these glucose-induced upregulations. Right panel: Protein levels of **(F)** FN, **(G)** COL4α1, **(H)** VEGF-A, **(I)** ET-1, and **(J)** p300 in cultured HMVECs as described for HRECs. Following the treatment of curcumin or its analogs in HMVECs incubated with HG, the protein markers exhibited downregulation [data expressed as a fold-change (mean ± SEM); normalized to NG; ^#^significance between NG vs. HG; *significance between HG vs. Treatment group; *^, #^*P* < 0.05, **^, *##*^*P* < 0.01, ***^, *###*^*P* < 0.001, and ****^, *####*^*P* < 0.0001; *n* = 6 from three independent experiments and performed in triplicates; n.s., not significant; NG (normal glucose) = 5 mM D-glucose; HG (high glucose) = 25 mM D-glucose; data analyzed by ANOVA followed by Tukey's *post-hoc*].

Although unique RNA alterations were observed in the endothelial cells following L2H21 or L50H46 treatment, we wanted to delineate whether similar alterations exist at the protein level. Therefore, we selected specific protein markers for examination (FN, COL4α1, VEGF-A, ET-1, and p300) in HRECs and HMVECs. Exposure of cells to HG showed that all proteins are increased in HRECs and HMVECs. Pre-treatment of HRECs with L2H21 or L50H46 prior to HG exposure prevented the increases in FN (Figure 2A; L2H21 = 36% reduction, L50H46 = 44% reduction), COL4α1 (Figure 2B; L2H21 = 40%, L50H46 = 58%), VEGF-A (Figure 2C; L2H21 = 59%, L50H46 = 71%), ET-1 (Figure 2D; L2H21 = 42%, L50H46 = 33%), and p300 (Figure 2E; L2H21 = 66%, L50H46 = 58%). Interestingly, curcumin was only able to reduce COL4α1 (34% reduction), VEGF-A (46%), and p300 (60%) proteins. Similarly, in HMVECs, L2H21 treatment significantly downregulated FN, COL4α1, VEGF-A, ET-1 and p300 proteins by approximately 39, 43, 75, 32, and 55%, respectively, as compared to HG (Figures 2F–J). L50H46 treatment also significantly decreased FN (33%), COL4α1 (50%), VEGF-A (53%), and p300 (78%) in HMVECs compared to HG. On the other hand, when curcumin-treated HMVECs were compared to HG, only protein markers for COL4α1, VEGF-A, and p300 were significantly reduced. Furthermore, we found statistically significant differences between L50H46 and curcumin treatments for FN expressions in HRECs (*P* = 0.0419); while, in HMVECs, differences existed between L2H21 and curcumin treatments for VEGF-A (*P* = 0.0464) and between L50H46 and curcumin treatments for p300 (*P* = 0.0368) proteins.

### Curcumin Analogs Reduced Oxidative DNA Damage *in vitro*

**Figure 3:**
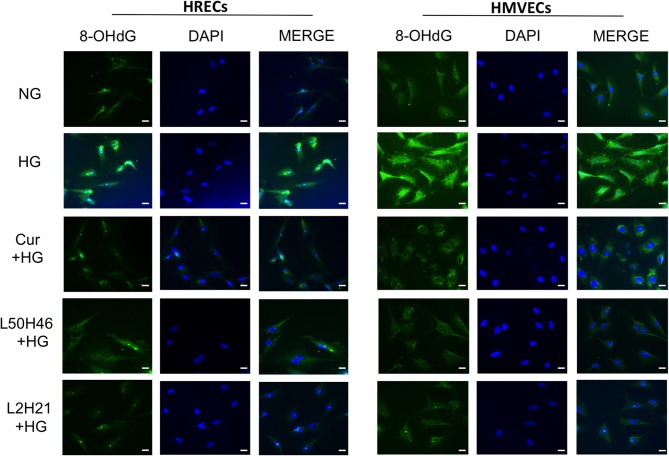
Effect of curcumin and its analogs on high glucose-induced oxidative DNA damage in HRECs and HMVECs. 8-OHdG staining was used to assess high glucose-induced oxidative DNA damage. Cells were exposed to HG for 48 h, with or without pre-treatment with curcumin or its analogs. The fluorescent images indicate that curcumin and its analogs decrease the level of glucose-induced oxidative DNA damage in both HRECs and HMVECs [original magnification at 20x; scale bar = 10 μm, same magnification for all images; NG (normal glucose) = 5 mM D-glucose; HG (high glucose) = 25 mM D-glucose; green stain = 8-OHdG and blue (DAPI) stain = nuclear counterstain; images were merged, and each image is representative of at least 3 separate experiments].

To investigate whether curcumin analogs modulate oxidative stress, we measured oxidative DNA damage by 8-OHdG immunofluorescence staining. As shown in Figure 3, HG increased oxidative DNA damage in both HRECs and HMVECs. Interestingly, pre-treatment of cells with curcumin, L2H21, or L50H46 prior to HG challenge attenuated increases in 8-OHdG immunoreactivity. These results suggest that curcumin analogs dampen glucose-induced oxidative DNA damage.

### Curcumin Analogs Reduce the Expressions of Pertinent Diabetes-Associated RNA Molecules in Target Organs of Diabetic Complications *in vivo*

**Figure 4:**
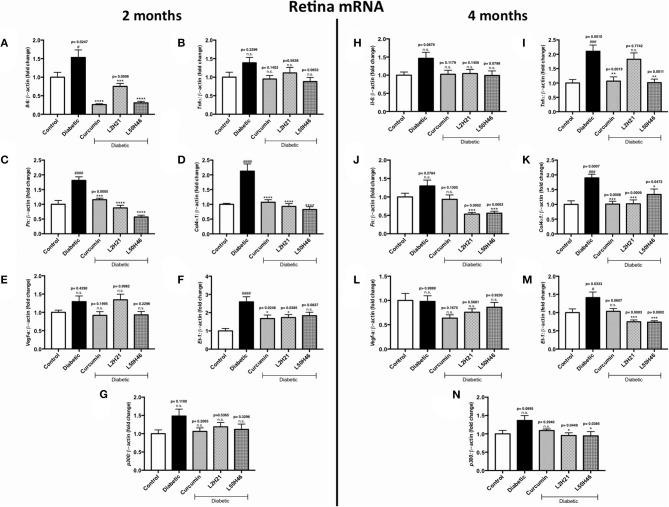
Effects of curcumin and its analogs on retinal transcripts at 2- and 4-months following induction of diabetes. Left panel: RT-qPCR analyses showing the upregulation of **(A)** *Il-6*, **(B)** *Tnf-*α, **(C)** *Fn*, **(D)** *Col4*α*1*, **(E)** *Vegf-a*, **(F)** *Et-1*, and **(G)** *p300* mRNAs in the retinal tissues of diabetic mice at 2 months. Administration of curcumin or its analogs produced differential RNA expressions in the retinal tissues of diabetic mice. Right panel: RT-qPCR analyses demonstrating relative increases of **(H)** *Il-6*, **(I)** *Tnf-*α, **(J)** *Fn*, **(K)** *Col4*α*1*, **(L)** *Vegf-a*, **(M)** *Et-1*, and **(N)** *p300* mRNAs in the retinal tissues of diabetic mice at 4 months. Treatment with curcumin or its analogs produced differential transcript expressions [data expressed as a ratio to β*-actin* (mean ± SEM); normalized to non-diabetic control; ^#^significance between Control vs. Diabetic; *significance between Diabetic vs. Treatment group; *^, #^*P* < 0.05, **^, *##*^*P* < 0.01, ***^, *###*^*P* < 0.001, and ****^, *####*^*P* < 0.0001; *n* = 6 animals/group; n.s., not significant; Control = non-diabetic controls; data analyzed by ANOVA followed by Tukey's *post-hoc*].

**Figure 5:**
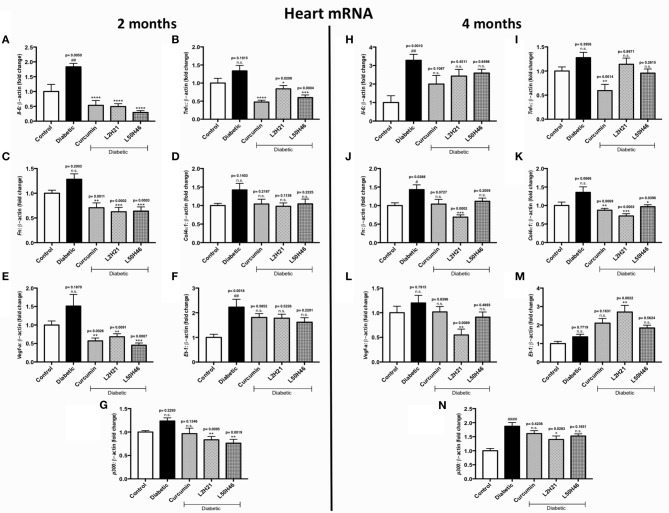
Effects of curcumin and its analogs on cardiac transcripts at 2 and 4 months. Left panel: RT-qPCR analyses showing increased mRNA levels of **(A)** *Il-6*, **(B)** *Tnf-*α, **(C)** *Fn*, **(D)** *Col4*α*1*, **(E)** *Vegf-a*, **(F)** *Et-1*, and **(G)** *p300* in the cardiac tissues of diabetic mice at 2 months. Administration of curcumin, L2H21, or L50H46 produced differential expressions of these transcripts in the cardiac tissues of diabetic mice. Right panel: RT-qPCR analyses of **(H)** *Il-6*, **(I)** *Tnf-*α, **(J)** *Fn*, **(K)** *Col4*α*1*, **(L)** *Vegf-a*, **(M)** *Et-1*, and **(N)** *p300* in cardiac tissues at 4 months. Treatment with curcumin or its analogs at this time point produced differential RNA expressions [data expressed as a ratio to β*-actin* (mean ± SEM); normalized to Control; ^#^significance between Control vs. Diabetic; *significance between Diabetic vs. Treatment group; *^, #^*P* < 0.05, **^, *##*^*P* < 0.01, ***^, *###*^*P* < 0.001, and ****^, *####*^*P* < 0.0001; *n* = 6 animals/group; n.s., not significant; Control = non-diabetic controls; data analyzed by ANOVA followed by Tukey's *post-hoc*].

**Figure 6:**
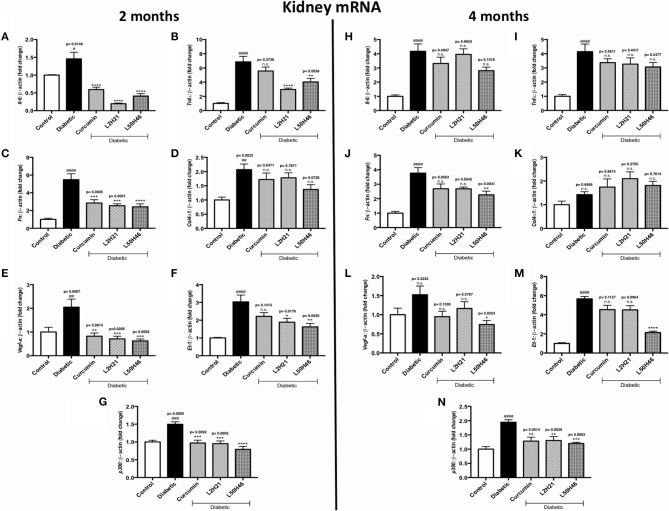
Effects of curcumin and its analogs on renal transcripts at 2 and 4 months. Left panel: RT-qPCR analyses demonstrating significant upregulations of **(A)** *Il-6*, **(B)** *Tnf-*α, **(C)** *Fn*, **(D)** *Col4*α*1*, **(E)** *Vegf-a*, **(F)** *Et-1*, and **(G)** *p300* mRNAs in the kidney tissues of diabetic mice at 2 months. Administration of curcumin or its analogs produced differential RNA expressions. Right panel: RT-qPCR analyses exhibiting relative upregulations of **(H)** *Il-6*, **(I)** *Tnf-*α, **(J)** *Fn*, **(K)** *Col4*α*1*, **(L)** *Vegf-a*, **(M)** *Et-1*, and **(N)** *p300* transcripts in kidney tissues at 4 months. Treatment with curcumin or its derivatives also produced differential RNA expressions in the diabetic mice kidneys [data expressed as a ratio to β*-actin* (mean ± SEM); normalized to Control; ^#^significance between Control vs. Diabetic; *significance between Diabetic vs. Treatment group; *^, #^*P* < 0.05, **^, *##*^*P* < 0.01, ***^, *###*^*P* < 0.001, and ****^, *####*^*P* < 0.0001; *n* = 6 animals/group; n.s., not significant; Control = non-diabetic controls; data analyzed by ANOVA followed by Tukey's *post-hoc*].

To determine whether similar transcript level reductions occur at the tissue level after L2H21 or L50H46 treatment, we examined tissues from diabetic mice treated with the analogs. When compared to non-diabetic controls, diabetic mice demonstrated increases in the panel of selected transcripts in the retina, heart, and kidney tissues in a time- and tissue-dependent manner (Figures 4–**6**). In the retina at both end points, most of the transcripts appeared to be downregulated following treatment with curcumin or its analogs when compared to untreated diabetic mice (Figures 4A–N). Specifically, at 2 months, significant decreases were observed in *IL-6* (Figures 4A), *FN* (Figure 4C), *COL4*α*1* (Figure 4D), and *ET-1* (Figure 4F) levels following curcumin or L2H21 treatment; whereas, L50H46 treatment significantly decreased *IL-6, FN*, and *COL4*α*1* transcript levels. Moreover, at the 4-month time point, significantly lower *TNF-*α (Figure 4I), *FN* (Figure 4J), *COL4*α*1* (Figure 4K), *ET-1* (Figure 4M), and *p300* (Figure 4N) levels were noted in the retinal tissues of L50H46-treated mice when compared to untreated diabetic controls. At this time point and compared to the same controls, L2H21-treated group only showed significant reductions in *FN* (59%), *COL4*α*1* (46%), *ET-1* (47%), and *p300* (30%); while, curcumin significantly reduced *TNF-*α (49%) and *COL4*α*1* (47%) transcript levels. Curcumin and its analogs were relatively comparable in impact for most of the transcripts at 2 and 4 months, with significances mainly observed for *IL-6* (curcumin vs. L2H21, *P* = 0.0443) and *FN* (L50H46 vs. curcumin, *P* = 0.0019) at 2 months, and *TNF-*α (L50H46 vs. L2H21, *P* = 0.0191, and curcumin vs. L2H21, *P* = 0.0316) at 4 months.

We then performed similar analyses and examined the effects of curcumin and its analogs on cardiac tissues. Administration of curcumin, L2H21, or L50H46 appeared to downregulate majority of the transcripts in cardiac tissues of diabetic mice (Figures 5A–L, **N**). In fact, when compared to untreated diabetic mice at 2 months, L2H21 and L50H46-treated mice had significantly lower *IL-6* (Figure 5A), *TNF-*α (Figure 5B), *FN* (Figure 5C), *VEGF-A* (Figure 5E), and *p300* (Figure 5G) levels; while, curcumin-treated mice also produced similar statistically significant reductions in the above markers, with the exception of *p300*. Furthermore, L2H21 treatment for a 4-month duration significantly diminished cardiac transcripts encoding *FN* (Figure 5J, 52% reduction), *COL4*α*1* (Figure 5K, 47% reduction), *VEGF-A* (Figure 5L, 54% reduction), and *p300* (Figure 5N, 25% reduction) when compared to untreated diabetic mice. While significant reductions in transcripts, compared to untreated diabetic mice, were observed for *TNF-*α (Figure 5I, ~54%) and *COL4*α*1* (~36%) following curcumin treatment and for *COL4*α*1* (~29%) following L50H46 treatment at 4 months. Interestingly, cardiac tissues from mice treated with curcumin or its analogs provoked the upregulation of *ET-1* at 4 months (Figure 5M)—with significant differences observed for L2H21 treatment as compared to untreated diabetic mice. Our results also show that curcumin and its analogs are comparable in the reduction of cardiac transcripts at 2 months. However, at the 4-month time point, significant differences in effects existed between curcumin and L2H21 treatments for *TNF-*α (*P* = 0.0129), and between L2H21 and L50H46 treatments for *FN* (*P* = 0.0402).

Lastly, we extended our analyses to kidney tissues. We found that when compared to untreated diabetic controls, *IL-6* (Figure 6A), *TNF-*α (Figure 6B), *FN* (Figure 6C), *VEGF-A* (Figure 6E), *ET-1* (Figure 6F), and *p300* (Figure 6G) transcripts were significantly dampened in renal cortical tissues after L2H21 or L50H46 treatment at 2 months of diabetes; while, curcumin treatment only demonstrated significant downregulations in *IL-6, FN, VEGF-A*, and *p300* transcripts. Moreover, at 4 months, L50H46 treatment significantly decreased *FN* (Figure 6J) and *VEGF-A* (Figure 6L) expressions by approximately 40 and 51%, respectively, when compared to untreated diabetic mice. As well, *p300* RNA levels were significantly reduced by curcumin (34%), L2H21 (33%), and L50H46 (38%) treatments at 4 months (Figure 6N). Of note, slight elevations in *COL4*α*1* transcripts were detected at 4 months following treatment with curcumin or its analogs (Figure 6K); however, these changes were not significant. L2H21 and curcumin treatments generated different response levels for *IL-6* (*P* = 0.0416) and *TNF-*α (*P* = 0.0082) at 2 months, while significances existed between treatments in *ET-1* expressions at 4 months (L50H46 vs. L2H21, *P* = 0.0001, and L50H46 vs. curcumin, *P* = 0.0001; Figure 6M).

### The Impact of Curcumin Analogs on Protein Levels of Diabetic Complications-Associated Pathways

To build on our *in vitro* results, we selected one marker each from the signaling pathways (TNF-α, COL4α1, ET-1, and p300) and examined the levels of these proteins in retinal, cardiac, and kidney tissues of mice. Similar to our findings *in vitro*, all proteins showed higher levels in untreated diabetic mice (Figures S3–S5). However, analysis of the effects of curcumin analogs showed differential responses at the 2 and 4-month time points. For example, in retinal tissues, significant decreases were observed in p300 expressions after L2H21 treatment at 2 months (Figure S3D; 48% reduction), and in TNF-α (Figure S3E; 89%) and COL4α1 (Figure S3F; 71%) expressions after L50H46 and curcumin treatments, respectively, at 4 months. Additionally, inter-treatment differences existed in retinal TNF-α (L50H46 vs. curcumin, *P* = 0.0315, and L50H46 vs. L2H21, *P* = 0.0023) and COL4α1 (curcumin vs. L50H46, *P* = 0.0480) at 4 months. Overall, treatment with curcumin, L2H21 or L50H46 had relatively comparable effects on the expression of these proteins in diabetic retinal tissues.

Moreover, compared to untreated diabetic mice, cardiac tissues of mice treated with curcumin or its analogs showed significantly reduced protein levels of TNF-α (Figure S4A), ET-1 (Figure S4C), and p300 (Figure S4D) at 2 months, and TNF-α (Figure S4E), COL4α1 (Figure S4F), ET-1 (Figure S4G), and p300 (Figure S4H) at 4 months. L2H21 and L50H46 treatments also showed differences when compared to curcumin for TNF-α at 2 months (L2H21 vs. curcumin, *P* < 0.0001, and L50H46 vs. curcumin, *P* < 0.0001); while, at 4 months, L50H46 treatments showed differences when compared to curcumin for TNF-α (*P* = 0.0010) and ET-1 (*P* = 0.0160). Nevertheless, similar to the results of retinal tissues, administration of curcumin or its analogs produced comparable reductions of proteins in cardiac tissues.

We next examined the protein levels in renal cortical tissues. When compared to kidneys harvested from diabetic mice, L2H21-treated mice only showed significant reductions in p300 proteins at 2 months (Figure S5D; 35% decrease); while, L50H46-treated mice showed decreases in TNF-α (Figure S5E; 70%) and p300 (Figure S5H; ~37%) proteins at 4 months. Alternatively, inter-treatment significance existed between L50H46 and L2H21 treatments for TNF-α at 4 months (*P* = 0.0141), whereas, the remaining markers did not show any differences between treatments.

Overall, at the protein level, our *in vitro* and *in vivo* findings suggest that intervention with curcumin or its analogs can induce general downregulations of key protein mediators involved in inflammation, fibrosis, and angiogenesis.

### Curcumin Analogs Prevent Cardiac and Renal Dysfunction in Diabetic Mice

Our next objective was to explore whether L50H46 or L2H21 treatments can improve the functional deficits characteristic of diabetic heart and kidney diseases. At 2 months, echocardiographic examination did not reveal decreases of E/A and E′/A′ ratios in untreated diabetic mice compared to non-diabetic controls (Figures S6A,B). While, at 4 months, reduced E/A ratios (27% reduction, *P* = 0.0040) were observed in diabetic mice (Figures S6F,G). However, when diabetic animals were treated with curcumin analogs, E/A and E′/A′ ratios were partially improved compared to untreated diabetic mice; in which, L2H21 and L50H46 treatments exhibited significant improvements in E/A ratios at 4 months (L2H21 improved E/A ratio by 35%, *P* = 0.0063, and L50H46 improved E/A ratio by 32%, *P* = 0.0083, when compared to untreated diabetic mice). Furthermore, increases in fractional shortening were observed in diabetic animals and treatment with curcumin, L2H21, or L50H46 showed partial reductions in fractional shortening at the time points (Figures S6C,H). As for quantifying cardiac hypertrophy, we used heart weight/tibia length measurements. Our findings demonstrated that increases in heart weight/tibia length ratios from the heart of diabetic animals were prevented by treatment with curcumin analogs, L50H46 and L2H21, after both 2 and 4 months (Figures S6D,I).

We further evaluated renal albumin excretion. Diabetic mice displayed significantly elevated urinary albumin excretion at 2 (*P* < 0.0001) and 4 (*P* = 0.0002) months compared to non-diabetic animals (Figures S6E,J). Treatment of mice with curcumin analogs showed reductions in urinary albumin excretion at both time points.

## Discussion

In the last few decades, research on the biological activities of curcumin has dramatically expanded. The ability of curcumin to act as a multifaceted agent makes its clinical use appealing. Despite the documented biological activities of curcumin, the therapeutic use of curcumin has posed certain challenges. For example, a study by Sharma et al. found that the administration of *Curcuma* extract had low oral bioavailability in patients with advanced colorectal cancer, since curcumin and its metabolites were only detectable in the feces of patients and not in the plasma, blood cells, or urine (21). Their findings are in keeping with additional animal studies that have documented the rapid metabolic clearance of curcumin (41–43). Stirred by these limitations, the development of new synthetic curcumin analogs becomes critical for improving the physicochemical properties of curcumin. Therefore, in the present study, we tested two novel curcumin analogs and explored the effects of these compounds on a number of pertinent molecules implicated in the pathogenesis of diabetic complications.

To explore and provide insights into the effects of L2H21 and L50H46, we first investigated these two novel mono-carbonyl analogs on select RNA and protein markers across two human endothelial cell types (HRECs and HMVECs) and three organ tissues that are commonly targeted in diabetes (retinal, cardiac, and renal). Our results in cultured cells show that the pretreatment of endothelial cells with curcumin and its analogs produce differential reductions in several RNA transcripts when compared to cells exposed to HG alone. Our data further shows that in HRECs, L50H46 exhibits stronger effects compared to curcumin. Whereas, L2H21-treated HMVECs showed more significantly reduced expressions for 4 out of the 7 RNA markers when compared to curcumin treatments. The differential responses seen in the two endothelial-cell types after curcumin analog treatment may suggest cell specificity. For example, in support of our observations, Khar et al. have shown that particular cancer cell lines exhibit differential apoptotic behavior in response to curcumin *in vitro*—suggesting that the effects of curcumin and its analogs may be dependent on the cell type (13). Similarly, when considering animal tissues, differential transcript expressions were also found across the three organs following curcumin, L2H21, and L50H46 administration, which allude to the complex nature of the interactions between these compounds and organ-specific cells. To further extend our gene expression findings, we examined the influence of these treatments on selected proteins *in vitro* and *in vivo*. Most of the proteins assayed followed similar patterns after curcumin, L2H21, or L50H46 treatment, in both cells and tissues of diabetic mice. Furthermore, through our echocardiographic analyses, we found that curcumin and its analogs were able to partially improve diastolic functioning and reduce fractional shortening. Diabetic mice treated with curcumin, L2H21, or L50H46 also exhibited decreased level of cardiac hypertrophy proxy and urinary albumin excretion when compared to untreated diabetic mice. In addition to our *in vivo* findings, findings from our 8-OHdG experiments strongly demonstrated that curcumin and its analogs reduce glucose-induced oxidative DNA damage *in vitro*. Collectively from these results, we believe that curcumin and its analogs may be capable of exerting therapeutic effects by modulating the RNA and protein machinery implicated in the pathogenesis of diabetic complications.

Inflammation is a key participant in the pathogenesis of diabetes and its complications (44). During inflammatory processes, TNF-α and IL-6 are upregulated (26–28, 34, 39, 45). Previous studies have found that curcumin supplementation can lower the levels of inflammatory cytokines in a number of different experimental models: ovaries of Wistar rats with polycystic ovary syndrome (46), HG-treated U937 monocytes and in the blood of diabetic (STZ-induced) Sprague Dawley rats (47), palmitate-stimulated 3T3-L1 adipocytes (48), and in LPS-activated macrophages (49). In keeping with these findings, our findings demonstrated similar reductions in inflammatory transcripts and proteins following treatment with curcumin and its analogs, which allude to the anti-inflammatory characteristics of these compounds. Although the exact mechanisms by which curcumin analogs reduce inflammatory responses in HG-challenged cells and tissues of diabetic mice remain unclear, one potential mechanism may be through the inhibition of toll-like receptor 4 (TLR4). TLR4, critical for innate immune responses against microbial agents, is also activated by a diverse group of endogenous ligands. We have previously shown that some curcumin analogs directly bind to a TLR4 co-receptor, myeloid differentiation protein 2 and inhibit TLR4 signaling (50, 51). TLR4 is implicated in diabetic nephropathy (52, 53), retinopathy (54), and cardiomyopathy (55). TLR4 inhibition also prevents HG-induced NF-κB activation and production of inflammatory cytokines (56). Furthermore, knockdown of TLR4 prevents HG-induced oxidative stress and induction of inflammatory cytokines in cultured podocytes (57) and vascular smooth muscle cells (58). Based on these findings, a future study may explore whether L2H21 and L50H46 also reduce inflammatory signaling in models of diabetic complications through the inhibition of TLR4.

In addition to inflammation, fibrosis is a critical response to diabetic tissue damage (59, 60). In fact, it is generally believed that enhanced extracellular matrix deposition and fibrosis are downstream of sustained inflammatory processes. A recent study by Chen and colleagues indicated that a curcumin derivative, J17, was able to significantly inhibit hyperglycemia-induced fibrosis in NRK-52E renal epithelial cells, H9C2 cardiomyoblast-like cells, and in the heart and kidneys of STZ-induced diabetic mice (61). More specifically, transforming growth factor-beta (TGF-β) and collagen 1 (COL1) RNA and protein levels were both found to be greatly reduced in the heart and kidney tissues of J17-treated diabetic mice. As well, compared to controls, significant decreases in the mRNA levels of *COL1, COL4*, and *TGF-*β were observed in HG-treated H9C2 and NRK-52E cells following the administration of J17. Moreover, Guo et al. demonstrated the ability of curcumin to significantly suppress the deposition of COL1 and COL3 in the cardiac tissues of diabetic rats, which was accompanied by reduced TGF-β1 production (62). The observations from their *in vivo* experiments were also confirmed with similar findings exhibited by HG-challenged human cardiac fibroblasts after curcumin administration. Nevertheless, in the present study and in keeping with our previous ones (12, 24, 27, 32, 33), we observed reductions in *FN* and *COL4* mRNAs and proteins in cultured cells and tissues following treatment with curcumin or its analogs. These observations corroborate the notion that curcumin and its analogs can influence proteins involved in fibrosis. It is likely that these effects are also mediated through suppression of inflammatory responses, as eluded to earlier.

Previous work from our laboratory documented that human umbilical vein endothelial cells (HUVECs) cultured in HG and treated with curcumin exhibit increased levels of *VEGF* and *ET-1* mRNAs (11). Moreover, work from other laboratories have generated similar results in which curcumin is capable of suppressing *VEGF-A* transcripts (62–65). Although we noted reductions in *VEGF-A* mRNA expressions, *ET-1* transcripts in cardiac tissues at 4 months demonstrated significant increases following L2H21 treatment when compared to untreated diabetic hearts. This unique finding is in keeping with our previous study where we observed similar upregulations of *ET-1* mRNA following curcumin treatment in cardiac tissues of STZ-induced diabetic rats and in HG-treated MVECs (17). Even though ET-1 protein levels at 4 months appeared to show the opposite response (significantly reduced) after the administration of curcumin or its analogs, it may be possible that a potential compensatory mechanism may be occurring (similar to the phenomenon observed in *COL4* RNA levels in kidney tissues at 4 months). Furthermore, it will be important to build on these observations and measure the levels of vasodilators; since, we know that the balance between vasoconstrictors (such as ET-1) and vasodilators (such as nitric oxide) is important for proper endothelium-dependent vasoregulation.

Both oxidative stress and inflammation converge on key transcription factors such as NF-κB to induce expression of adhesion molecules, inflammatory cytokines, or cause changes to cell growth and differentiation (66–68). Upon activation and binding to response elements of promoters, NF-κB requires p300/CBP complex for chromatic remodeling and gene expression (69). From our findings and in accordance with past studies (11, 12, 19), hyperglycemic environments significantly upregulated p300 RNA and protein expressions, which were accompanied with elevated vasoactive, inflammatory, and pro-fibrotic genes. We have also previously shown that p300-mediated histone acetylation can subsequently activate several transcription and vasoactive factors under hyperglycemic conditions, which can negatively affect vascular structure and function (11, 12). Specifically, we have demonstrated that HG conditions can promote p300 binding to the promoter regions of *ET-1* and *FN* genes and p300 can also regulate NF-κB activity via poly (ADP-ribose) polymerase (PARP) activation (11, 12). Curcumin has already been shown to directly inhibit the enzymatic activity and concentrations of p300 (70, 71), which subsequently attenuates the induction of inflammatory cytokines, vasoactive factors and ECM protein productions in various target cells and organs (11, 19, 72)—confirming the trends observed for curcumin and its analogs in the present study. On a similar note, another curcumin analog, known as C66, has been shown to prevent diabetic nephropathy in mice by inhibiting glucose-induced activation of the c-Jun N-terminal kinase pathway and p300 histone acetyltransferase activity, ultimately preventing the upregulation of pro-fibrotic cytokines (73). Nevertheless, additional p300-based studies are warranted for understanding the precise mechanisms behind the L2H21 and L50H46 compounds.

Our main objective was to determine the activity of curcumin analogs on key molecular and functional alterations associated with diabetic complications. This was essentially spurred from a vast amount of previous studies showing therapeutic effects for curcumin. However, it is well-known that curcumin bioavailability is a hindrance. One alternative is to develop analogs, which offer more favorable pharmacokinetic profiles, as performed in this study. We used relatively low doses of the mono-carbonyl curcumin analogs in our animal experiments. Both L2H21 and L50H46 treatments were able to demonstrate comparable effects on transcript and protein levels relative to curcumin. We also used a significantly higher dose of curcumin and its analogs in our *in vivo* toxicity studies and found no significant cytotoxic effects. Therefore, based on our data, these analogs are appealing candidates for further evaluation in preclinical studies.

## Data Availability Statement

All datasets generated for this study are included in the article/Supplementary Material.

## Ethics Statement

All animal procedures were approved by the Animal Care and Use Committees of University of Western Ontario, London, Ontario, Canada, and Wenzhou Medical University, Zhejiang, China, and the experiments were performed in accordance with The Guide for the Care and Use of Laboratory Animals (NIH Publication 85-23, revised in 1996).

## Author Contributions

SB, GL, LC, SCha, and ZK: experimental conception and design. SB, SChe, and BF: performed the experiments. SB, SChe, GL, BF, LC, SCha, and ZK: reagents, materials, analysis tools contribution. SB, SCha, and ZK: wrote the manuscript.

### Conflict of Interest

The authors declare that the research was conducted in the absence of any commercial or financial relationships that could be construed as a potential conflict of interest.
