# Supplementary material for: Curcumin Analogs Reduce Stress and Inflammation Indices in Experimental Models of Diabetes
Source: Front Endocrinol (Lausanne). 2019 Dec 18;10:887. doi: 10.3389/fendo.2019.00887 (PMC6930691; doi:10.3389/fendo.2019.00887)

## Slide 1
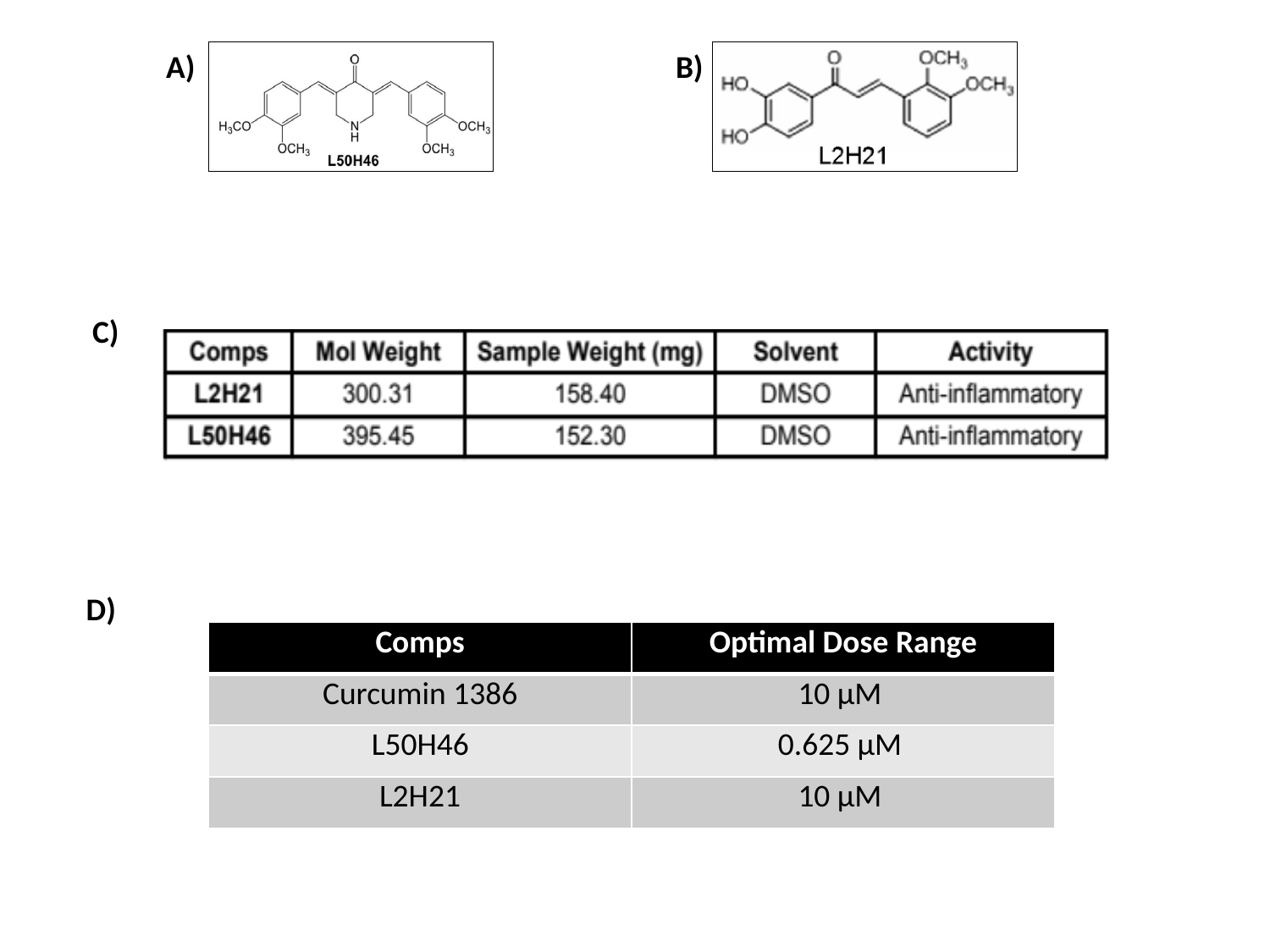

A)
B)
C)
D)
| Comps | Optimal Dose Range |
| --- | --- |
| Curcumin 1386 | 10 μM |
| L50H46 | 0.625 μM |
| L2H21 | 10 μM |

## Slide 2
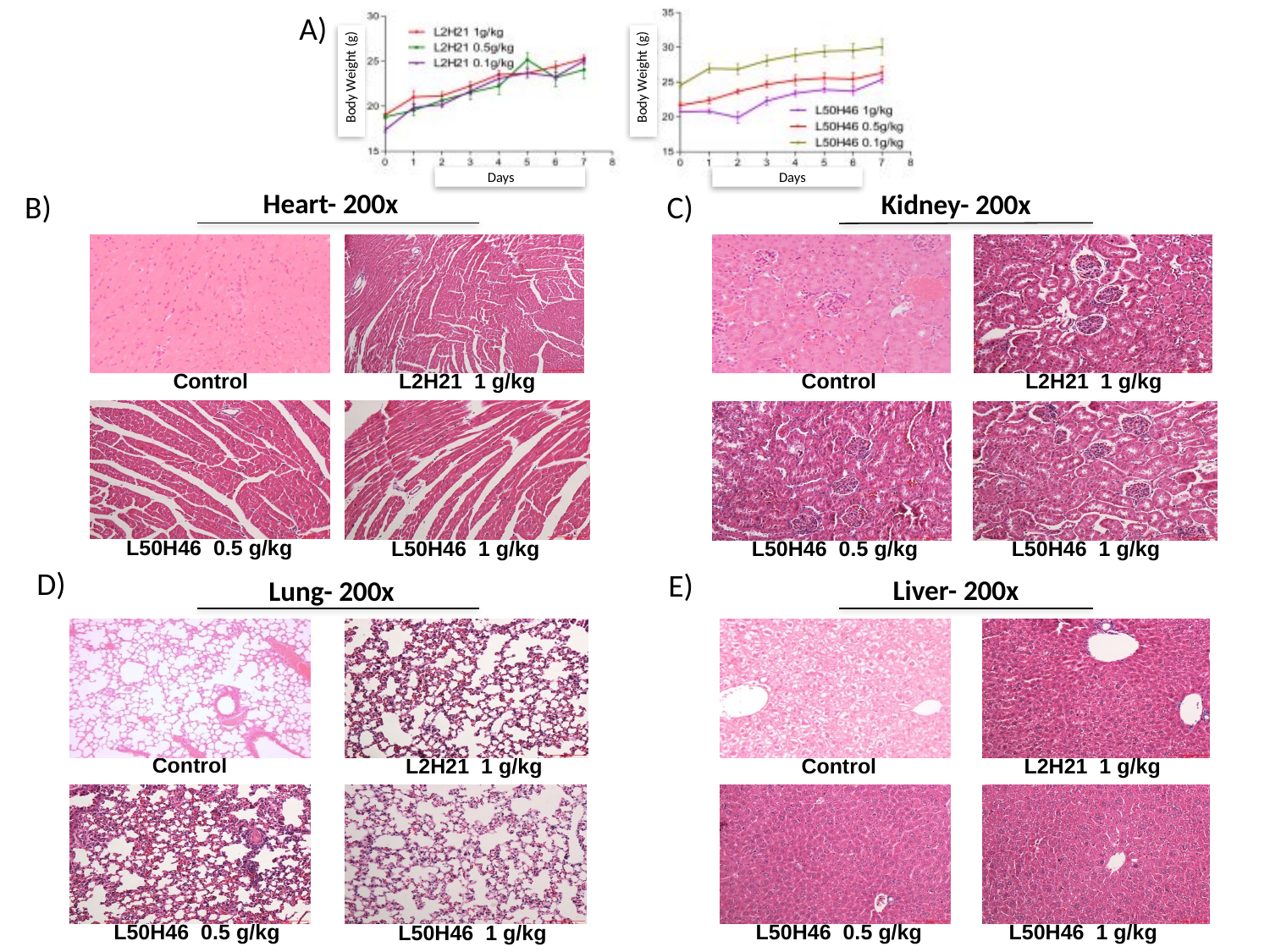

A)
Body Weight (g)
Body Weight (g)
Days
Days
Heart- 200x
C)
Kidney- 200x
B)
Control
Control
L2H21 1 g/kg
L2H21 1 g/kg
L50H46 0.5 g/kg
L50H46 1 g/kg
L50H46 0.5 g/kg
L50H46 1 g/kg
D)
E)
Liver- 200x
Lung- 200x
Control
Control
L2H21 1 g/kg
L2H21 1 g/kg
L50H46 0.5 g/kg
L50H46 1 g/kg
L50H46 0.5 g/kg
L50H46 1 g/kg

## Slide 3
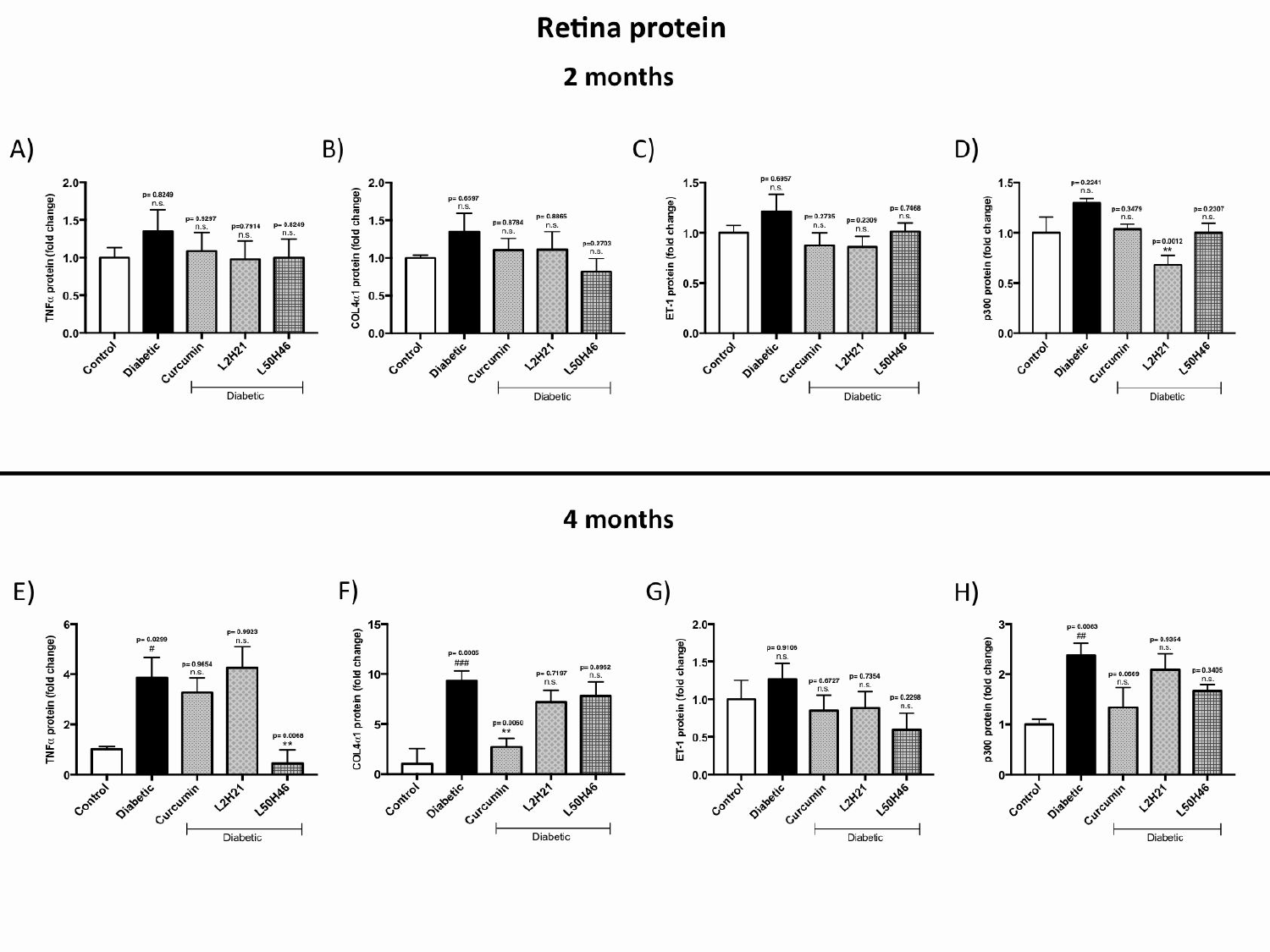

## Slide 4
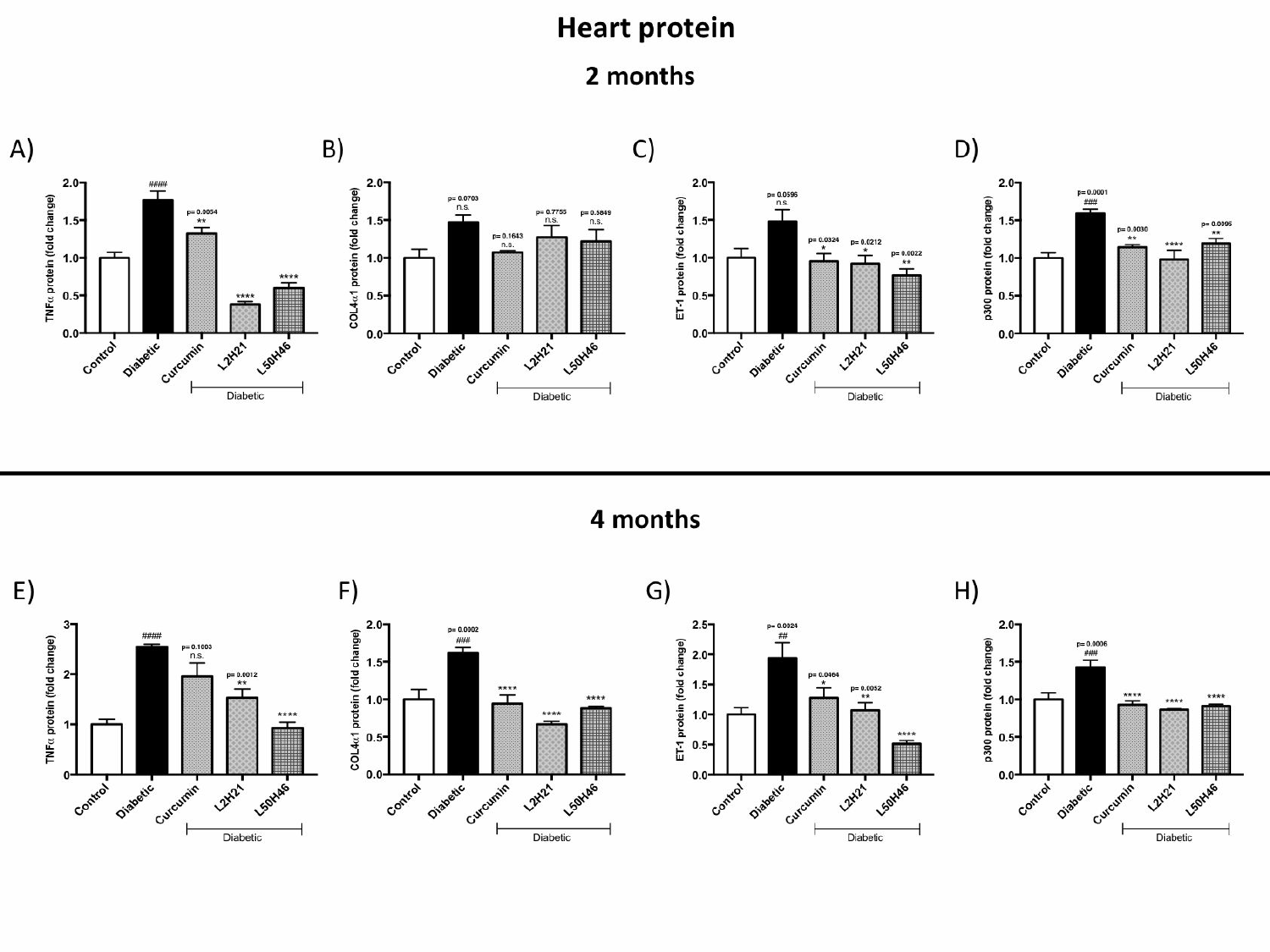

## Slide 5
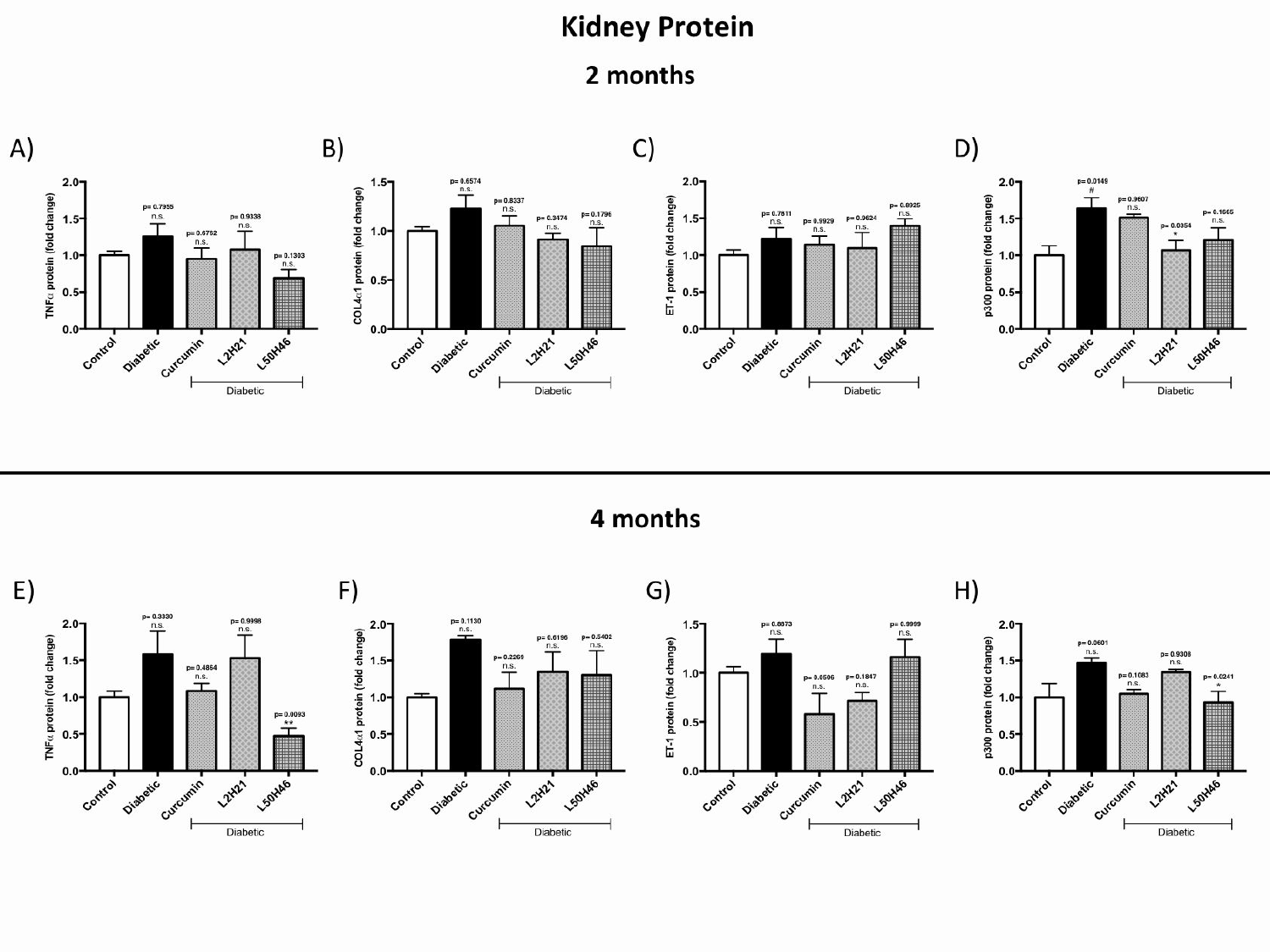

## Slide 6
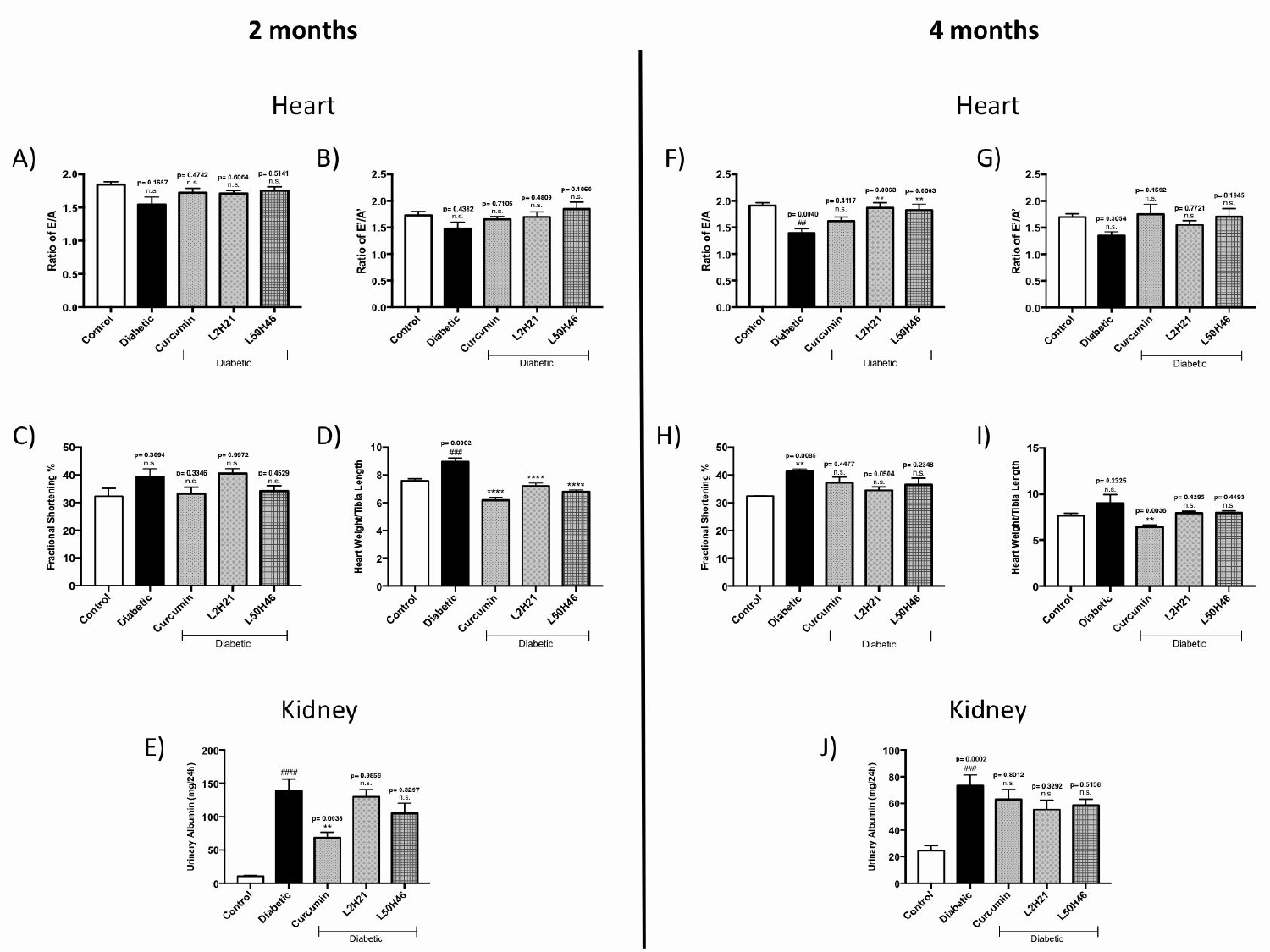

## Slide 7
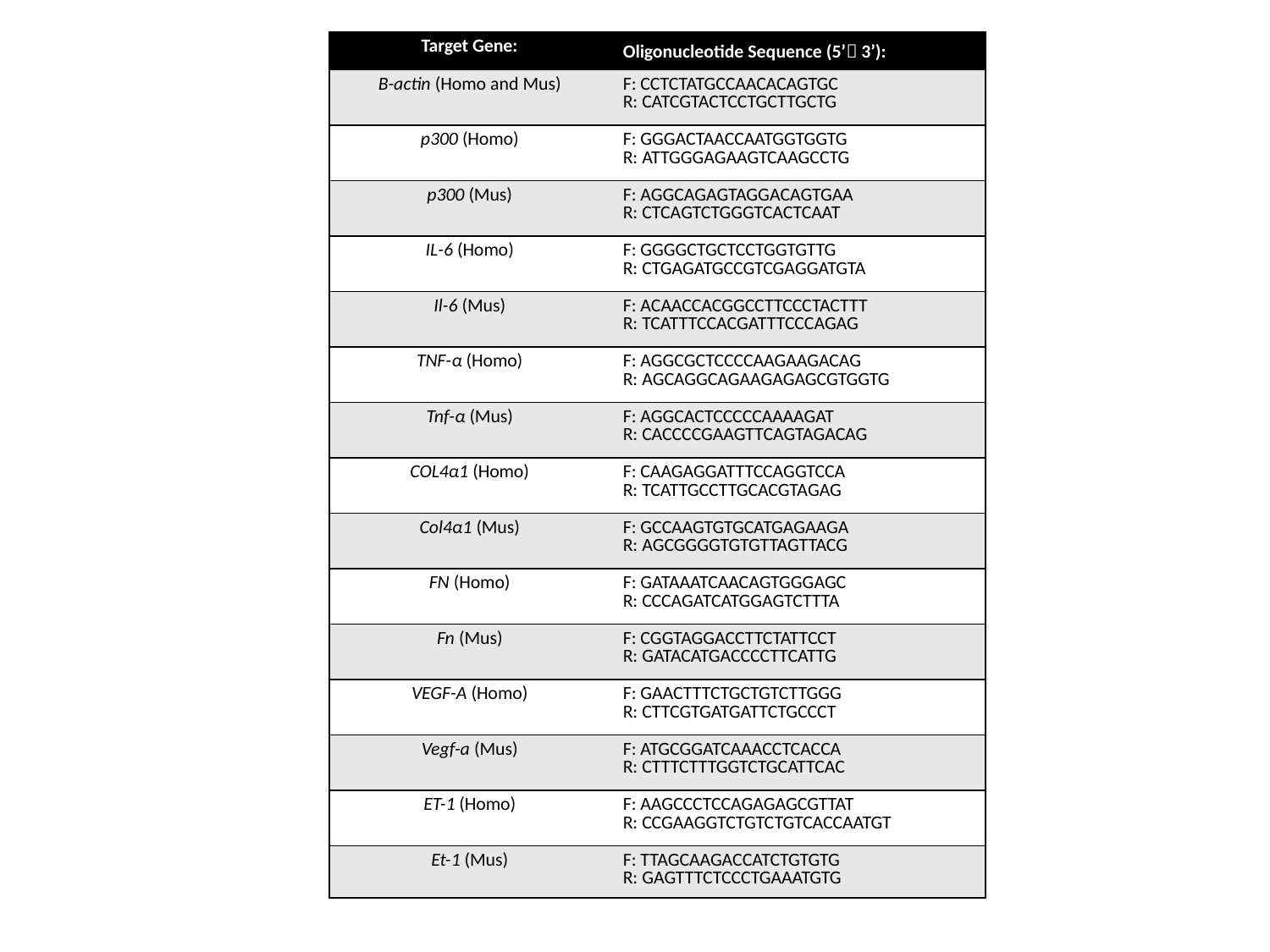

| Target Gene: | Oligonucleotide Sequence (5’ 3’): |
| --- | --- |
| B-actin (Homo and Mus) | F: CCTCTATGCCAACACAGTGC R: CATCGTACTCCTGCTTGCTG |
| p300 (Homo) | F: GGGACTAACCAATGGTGGTG R: ATTGGGAGAAGTCAAGCCTG |
| p300 (Mus) | F: AGGCAGAGTAGGACAGTGAA R: CTCAGTCTGGGTCACTCAAT |
| IL-6 (Homo) | F: GGGGCTGCTCCTGGTGTTG R: CTGAGATGCCGTCGAGGATGTA |
| Il-6 (Mus) | F: ACAACCACGGCCTTCCCTACTTT R: TCATTTCCACGATTTCCCAGAG |
| TNF-α (Homo) | F: AGGCGCTCCCCAAGAAGACAG R: AGCAGGCAGAAGAGAGCGTGGTG |
| Tnf-α (Mus) | F: AGGCACTCCCCCAAAAGAT R: CACCCCGAAGTTCAGTAGACAG |
| COL4α1 (Homo) | F: CAAGAGGATTTCCAGGTCCA R: TCATTGCCTTGCACGTAGAG |
| Col4α1 (Mus) | F: GCCAAGTGTGCATGAGAAGA R: AGCGGGGTGTGTTAGTTACG |
| FN (Homo) | F: GATAAATCAACAGTGGGAGC R: CCCAGATCATGGAGTCTTTA |
| Fn (Mus) | F: CGGTAGGACCTTCTATTCCT R: GATACATGACCCCTTCATTG |
| VEGF-A (Homo) | F: GAACTTTCTGCTGTCTTGGG R: CTTCGTGATGATTCTGCCCT |
| Vegf-a (Mus) | F: ATGCGGATCAAACCTCACCA R: CTTTCTTTGGTCTGCATTCAC |
| ET-1 (Homo) | F: AAGCCCTCCAGAGAGCGTTAT R: CCGAAGGTCTGTCTGTCACCAATGT |
| Et-1 (Mus) | F: TTAGCAAGACCATCTGTGTG R: GAGTTTCTCCCTGAAATGTG |

## Slide 8
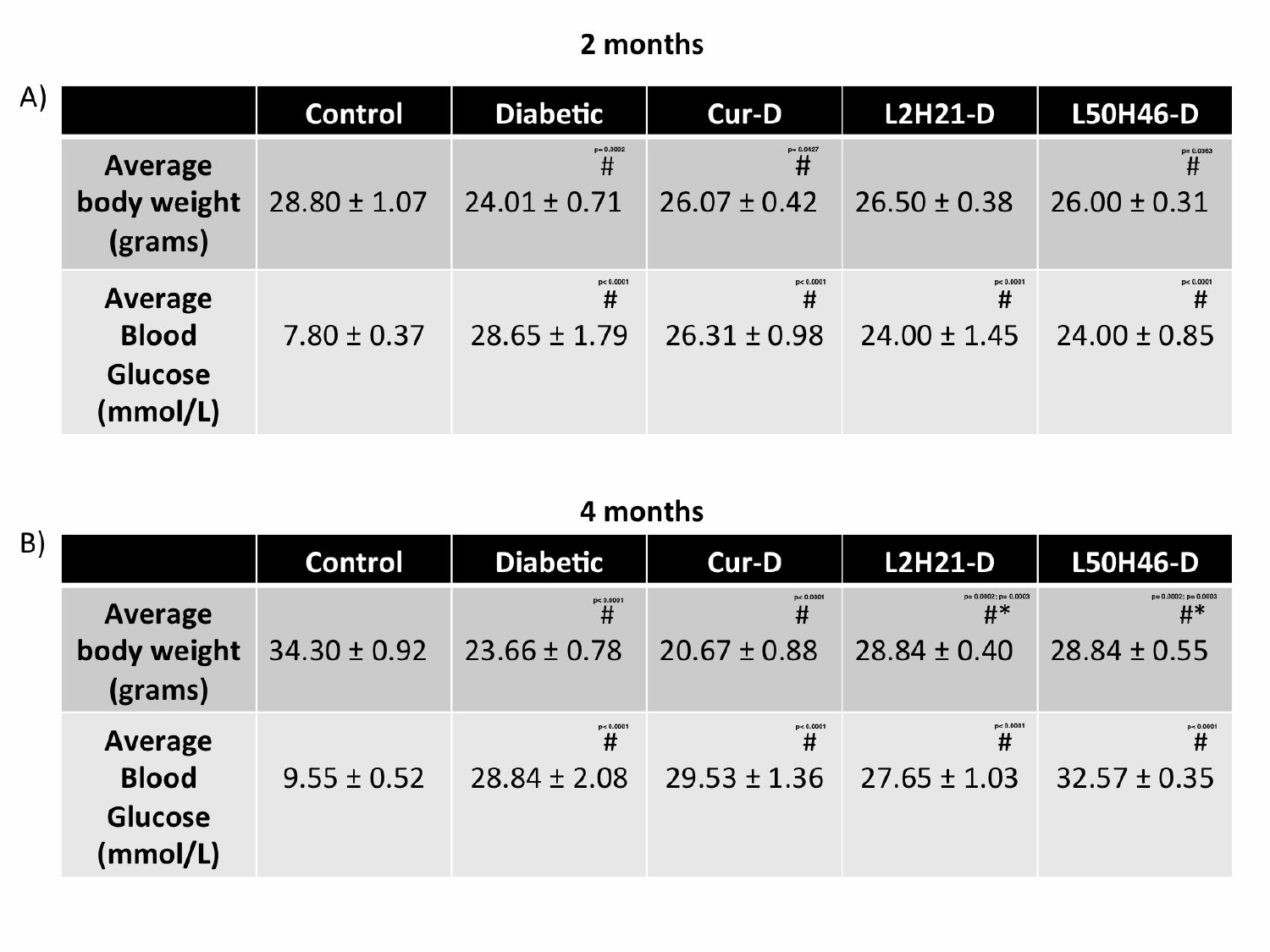

Supplement: Supplementary file 1 [file Data_Sheet_1.zip › Supplementary figures and tables only.pptx]
